# Ethylene responsive transcription factor ERF109 retards PCD and improves salt tolerance in plant

**DOI:** 10.1186/s12870-016-0908-z

**Published:** 2016-10-06

**Authors:** Ahmed Bahieldin, Ahmed Atef, Sherif Edris, Nour O. Gadalla, Hani M. Ali, Sabah M. Hassan, Magdy A. Al-Kordy, Ahmed M. Ramadan, Rania M. Makki, Abdulrahman S. M. Al-Hajar, Fotouh M. El-Domyati

**Affiliations:** 1 Department of Biological Sciences, Faculty of Science, King Abdulaziz University (KAU), P.O. Box 80141, Jeddah, 21589 Saudi Arabia; 2 Princess Al-Jawhara Al-Brahim Centre of Excellence in Research of Hereditary Disorders (PACER-HD), Faculty of Medicine, King Abdulaziz University (KAU), Jeddah, Saudi Arabia

**Keywords:** Knockdown, VIGS, Knockout, T-DNA, Over-expression, sqRT-PCR

## Abstract

**Background:**

The ultimate goal of this work was to detect the role of transcription factors (TFs) concordantly expressed with genes related to programmed cell death (PCD) during PCD and salt stress. This work was based on the hypothesis that TFs and their driven genes likely co-express under different stimuli. The conserved superfamily ethylene responsive factor (AP2/ERF) draw attention of the present study as it participates in the response to biotic and abiotic stimuli as well as to program cell death (PCD).

**Results:**

RNA-Seq analysis was done for tobacco (*N. benthamiana*) leaves exposed to oxalic acid (OA) at 20 mM for 0, 2, 6, 12 and 24 h to induce PCD. Genes up-regulated after 2 h of OA treatment with known function during PCD were utilized as landmarks to select TFs with concordant expression. Knockdown mutants of these TFs were generated in tobacco via virus induced gene silencing (VIGS) in order to detect their roles during PCD. Based on the results of PCD assay, knockout (KO) T-DNA insertion mutants of Arabidopsis as well as over-expression lines of two selected TFs, namely ERF109 and TFIID5, analogs to those in tobacco, were tested under salt stress (0, 100, 150 and 200 mM NaCl).

**Conclusions:**

Results of knockdown mutant tobacco cells confirmed the influence of these two TFs during PCD. Knockout insertion mutants and over-expression lines indicated the role of ERF109 in conferring salt tolerance in Arabidopsis.

**Electronic supplementary material:**

The online version of this article (doi:10.1186/s12870-016-0908-z) contains supplementary material, which is available to authorized users.

## Background

PCD has a major role in mediating plant adaptive responses to harsh conditions. Hypersensitive response (HR) represents the most common type of PCD in plants basically as a result of pathogen attack [1]. Several reports indicated that PCD also occurs in response to various abiotic stresses including salinity [2–4]. Salt stress was reported to elevate reactive oxygen species (ROS) levels, thus, can induce PCD [5]. Hence, we hypothesized that retardation of PCD machinery might result in delayed response to salt stress in plant. Salt-induced PCD is affected by ion disequilibrium resulted during Na^+^/K+ exchange [6]. The latter process results in the induction of hydroxyl radicals that regulate several PCD-related proteins, ex., Bcl-2. This protein is antiapoptotic and represses the vacuolar processing enzymes (VPE), by modulating ion fluxes. Bcl-2 encoding gene also reduces K+ efflux under salt stress, hence, retards PCD [7]. The protein also interacts with Bax1 (BI-1) protein to block its action in inducing PCD [8]. In addition, Zhang et al. [9] indicated that expression of genes encoding the two cysteine protease inhibitors, e.g., AtCYSa and AtCYSb, improves salt tolerance in *Arabidopsis*.

A conserved TF superfamily, namely APETALA2/Ethylene Responsive Factor (AP2/ERF), has a special attraction in the plant kingdom as its members are involved in vital biological processes during growth and development and in the response to several biotic and abiotic stimuli. Therefore, TFs of this family were valuable targets for genetic transformation and plant breeding programs. However, the response to the growth regulator ethylene is not a universal feature of this superfamily [10, 11]. The family is subdivided in *Arabidopsis thaliana* according to the similarity of its AP2/ERF binding domain into two main groups, the dehydration-responsive element binding-proteins (DREBs) and the ERFs. Little information is available for the DNA binding properties of AP2 proteins, although they are known to interact with flowering-regulatory genes [12]. Several DREB members bind to an A/GCCGAC element existing upstream of genes responding to ABA, drought and cold [13]. ERF members bind specifically to another element, e.g., AGCCGCC or GCC-box upstream of genes responding to ethylene, pathogens and wounding [14]. DNA-binding affinities of members of this family are highly diversified to accommodate the plethora of responses to external stimuli [15–18]. Very little so far is known for the participation of ERF in PCD as only one member of this large gene family in *Nicotiana umbratica*, namely MACD1, was proven to positively regulate factors affecting PCD triggered by phytotoxin AAL [19]. The authors also triggered PCD in Arabidopsis using a structural analog of AAL, namely fumonisin B1 (FB1), and proved that another member, namely ERF102, participates in PCD. Overall, AP2/ERF superfamily genes were characterized in plant and proven to respond to cold, salt and drought stresses, and recommended in *Brassica* to be utilized *via* transgenic technology to improve tolerance to such adverse conditions [20].

The present study was based on the hypothesis that TFs (ex., ERF) and their driven genes likely co-express under different stimuli. PCD-related genes were used as landmarks to detect co-expressed TFs that might regulate their expression in tobacco (*N. benthamiana*), hence, detect influence of their analogs in Arabidopsis under salt stress. The overall data indicated the involvement of a TF of ERF family, namely ERF109, in cell death and salt tolerance.

## Methods

### Materials

Wild-type (WT) tobacco (*Nicotiana benthamiana*) plants (provided by Professor Gregory Martin, Plant Pathology and Plant-Microbe Biology, Boyce Schulze Downey Research Chair, Boyce Thompson Institute, Cornell University, Tower Road, Ithaca, NY 14853-1801, USA) were grown from seed in a growth room 22/20 °C ± 2 °C day/night temperatures with 16-h photoperiod. *Agrobacterium tumefaciens* and *Escherichia coli* strains were grown in LB medium at 30 and 37 °C, respectively. Ampicillin was used at 100 μg/ml, while at 50 and 100 μg/ml for kanamycin and rifampicin, respectively. Arabidopsis WT (Colombia) as well as the T-DNA insertion (SALK_150614 and SALK_021380) and over-expression (CS212871 and CS872747) lines of the two TFs, e.g., ERF109 and TFIID5, respectively, were provided by the SALK Institute, Genomic Analysis Laboratory (SIGnAL). The lines were grown in appropriate selective medium and screened for homozygosity by PCR. Sequences of primers and PCR conditions are available in Arabidopsis database (http://signal.salk.edu/tdnaprimers.2.html).

### RNA-Seq analysis

RNA-Seq was done and analyzed for RNAs extracted from leaf discs of 7-week-old tobacco (*N. benthamiana*) WT plants treated with oxalic acid (OA) at 20 mM for 0, 2, 6, 12 and 24 h to induce PCD as indicated earlier [8]. RNA samples were deep sequenced at BGI, China, which generated over 100 million reads per sample. Raw reads were filtered and aligned (≥2 mismatches) to the *N. benthamiana* draft genome (ftp.solgenomics.net/genomes/Nicotiana_benthamiana/annotation/Niben.genome.v0.4.4.transcripts.annotated.fasta). RSEM v1.1.6 was used to estimate the relative abundances read counts and utilized Bowtie aligner (Bowtie v0.12.1) to map the reads against assembled transcripts. Expected read counts were used as resources to differential expression analysis by EdgeR (version 3.0.0, R version 2.1.5). The unmapped sequences were re-aligned against the contigs collectively *de novo* assembled using the Trinity RNA-Seq Assembly package (r2013-02-25). Blastx was performed against the NCBI non-redundant protein database with an E-value cut off of 1e^-5^ in order to detect proteins with TF-related domain(s). Fold change (FC) values of DE transcripts were calculated against the published tobacco *actin* (*Nbactin*) as the house-keeping gene and FC of ≥ 5 was selected for further analysis. Then, significant Pearson correlation through permutation analysis was determined. The resulting clusters were refined visually and analyzed for GO terms using Blast2GO (http://www.blast2go.org/). To validate RNA-Seq data, semi-quantitative (sq) RT-PCR of selected TFs was done (data provided upon request).

### Construction of virus induced gene silencing (VIGS) lines and PCD assays

VIGS lines of selected TFs were generated in 4-week-old tobacco (*N. benthamiana*) seedlings as previously described [21]. Selection was based on the expression patterns and the co-expression data of TFs with PCD-related genes used as landmarks. Primers used in constructing the gateway compatible pTRV2 vectors [22] of different genes were synthesized. Empty pTRV vectors were provided by Professor Gregory Martin, Cornell University, USA). Then, spreading of the TRV virus in the newly emerged leaves was detected visually via the use of pTRV2-GFP construct [23] in transforming tobacco. Visualization of GFP in the transgenic plant was done by illumination with longwave (100 W) ultraviolet lamp. Efficiency of VIGS was detected via the use of TRV2-PDS [24]. The construct was used to knockdown *PDS* (*phytoene desaturase*) gene towards the generation of photo-bleaching in the newly developed leaves.

Leaf discs of WT and VIGS lines of tobacco (*N. benthamiana*) plants were obtained from 7-week-old plants using a 10-mm-diameter cork borer and discs were submerged in OA (Sigma-Aldrich) at 20 mM for 24 h. Then, scoring of cell death in VIGS of different lines was done quantitatively by Evans blue assay [25] and qualitatively by DNA laddering [26]. Expression of three selected TFs in tobacco VIGS lines was validated via qRT-PCR with WT and VIGS line with empty pTRV2 used as controls. For each sample, 2 μg of total RNA was used to synthesize first-strand cDNA with oligo(dT) using Revert Aid Premium Reverse Transcriptase (Thermo Scientific™ cat. no. EP0451). qRT-PCR was performed with gene-specific primers to amplify 190–199 bp (designed by GenScript Real-time PCR Primer Design, www.genscript.com). Templates were normalized to amplify 196 bp fragment of the tobacco *actin* or *Nbactin*, used as the reference gene. qRT-PCR was done in a total of 25-μl volume containing 1 μl cDNA, 12.5 μl 2 x BIO-RAD iQTMSYBR@GreenSupermix, 0.75 μl ROX reference dye (1:500 diluted), 1 μl 500 nM of each primer. All reactions were performed in triplets and run on a Mx3005P QPCR System (Stratagene) using the following conditions: 5 min at 95 °C, 40 cycles of 30s at 95 °C, 60s at 55–56 °C, 20s at 72 °C and 72 °C (overnight). PCR products were examined by melt curve analysis. Amplicons generated from *Nbactin* gene reached saturation at cycles between 18 and 21. Expression levels of TF genes relative to *Nbactin* gene were calculated using MxPro QPCR Software v4.10 package, which compares reaction takeoff points (cycle number). Relative mRNA abundance was estimated as previously described [27]. Knockdown of the other TFs in tobacco VIGS lines as well as knockout and over-expression of selected TFs in Arabidopsis were also proven via sqRT-PCR. Amplification was done with the conditions indicated earlier [8].

### Salt stress experiment for Arabidopsis T-DNA insertion knockout and over-expression lines

Based on PCD assays results, two salt stress experiments at 0, 100, 150 and 200 mM NaCl were conducted to detect the performances of T-DNA insertion knockout and over-expression lines of selected TFs as compared to the WT control (Col). Salt stress experiments were conducted with growth conditions indicated earlier [8]. The first experiment was conducted in order to detect germination percentages of seeds left to germinate on different salt treatments and scores were made at day 6, where no further seed germination can take place. The second experiment was conducted for 2-week-old seedlings left to grow on different salt treatments for two more weeks and measurements were made for root length (mm), number of leaves per plant and the rosette area (cm^2^).

### Statistical analyses

PCD assay and salt stress experiment were designed in randomized complete blocks with three replicates. Statistical analyses of different experiments were performed following the procedure outlined by Gomez and Gomez [28] and multiple comparisons were performed following Duncan’s New Multiple Range test [29].

## Results

### Transcription factors co-expressed with PCD-related genes

Based on our previous investigation [8], PCD-related genes existing in 16 clusters (Additional file 1: Table S1 & Additional file 2: Figure S1) of up-regulation after 2 h exposure to OA were detect, of which 23 genes were used as landmarks to detect concordantly expressed TFs. The results indicated that 31 TFs were concordantly expressed with the selected PCD-related genes in 10, out of the 16, clusters (Additional file 3: Table S2 and Additional file 4: Figure S2). These TFs mainly belong to four TF families, namely ERF (ABR1, ERF4, ERF5, ERF109), MYB (MYB305 and MYB306), WRKY (WRKY23, WRKY40, WRKY48, WRKY53 and WRKY70) and NAC (NAC01 and NAC010). They also include GTE8, DOF zinc finger, BED zinc, CPRF2, bHLH137, TFIID5, CRF4, SPT20 and TGA7. TFs detected in the four families were mainly cited to have roles during development or during biotic and/or abiotic stresses.

### Effects of OA on tobacco plants knocked down in selected TFs via VIGS

**Fig. 1:**
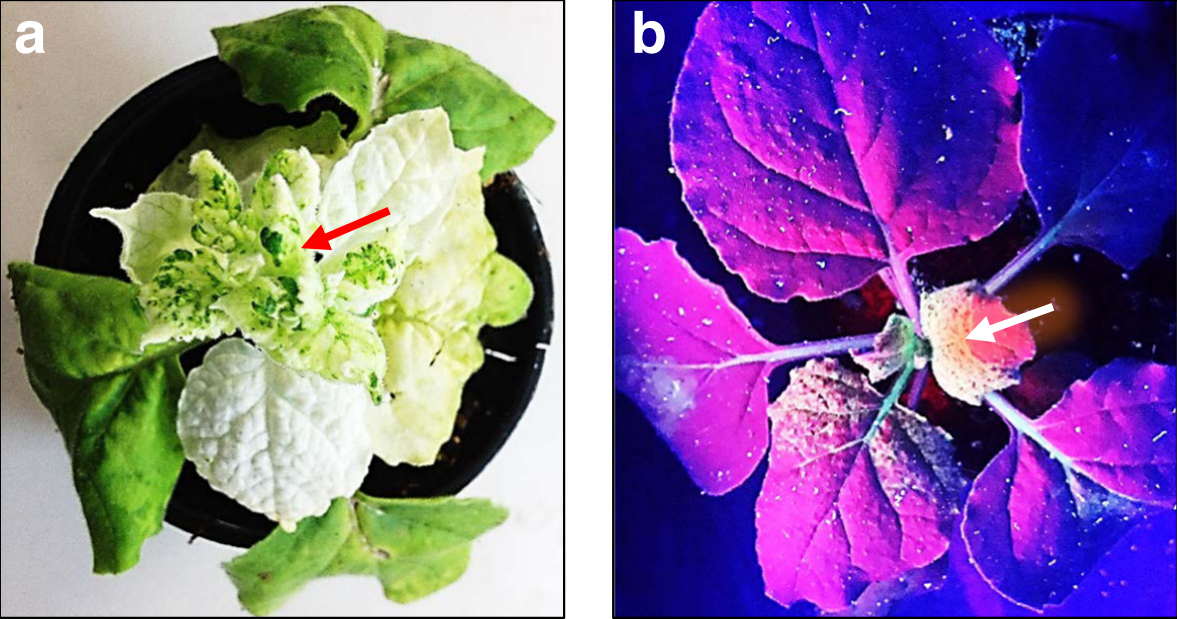
Silencing of the *PDS* gene to cause photo-bleaching (**a**) and over-expression of GFP (**b**) in tobacco (*N. benthamiana*) plants to prove the incidence of VIGS. Photographs were taken 21 days after infiltration. Arrows indicate the newly developed leaves with gene knocked down, e.g., *PDS* (**a**) or expressed, e.g., *GFP* (**b**) via VIGS

**Fig. 2:**
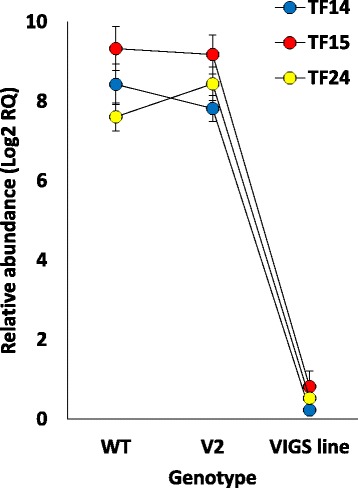
qRT-PCR analysis of relative transcript abundance of the three TFs (e.g., T14, T15 and T24) in their corresponding VIGS lines as compared to those in tobacco wild type (WT) and VIGS line with empty pTRV2 (V2) plants. The three TFs were induced 2 h after oxalic acid (OA) treatment. The *Nbactin* gene was used as the house-keeping control. Gene codes refer to those indicated in Additional file 3: Table S2. Data were statistically analyzed as outlined by Gomez and Gomez [28] and multiple comparisons were made following the Duncan’s New Multiple Range test [29]

Primers used in constructing pTRV2 for generating VIGS lines and in detecting levels of gene expression of different VIGS lines are shown in Additional file 5: Table S3. No morphological differences in terms of phenotype or growth performance were detected among the recovered VIGS lines, on one hand, or between the VIGS lines and WT plants, on the other hand. To morphologically detect the efficiency of VIGS and spreading of the virus, *PDS* (*phytoene desaturase*) gene was knocked down via VIGS and *GFP* gene was expressed in WT tobacco. The results indicated silencing (or photo-bleaching) of the *PDS* gene in its VIGS line and expression of *GFP* gene in the new leaves of transformed plants 21 days after infiltration (Fig. 1). Bright color refers to transformed cells expressing GFP, while violet color refers to non-transformed cells. The results of qRT-PCR and sqRT-PCR indicated that a number of 16, out of 31, tobacco TFs were successfully knocked down in tobacco VIGS lines (Fig. 2 & Additional file 6: Figure S3, respectively). Levels of expression in replicates of VIGS lines generated for each gene are uniform (data not shown).

Cell death after 24 h due to the treatment with OA in tobacco WT leaf discs was proven using Evans blue staining and DNA laddering (Additional file 7: Figure S4). The results of PCD assay either induced or repressed due to the knockdown of TFs in VIGS lines are shown in Table 1. The results indicated that VIGS lines of four TF genes responded differently as compared to the other VIGS lines as well as WT and VIGS with empty pTRV2. One of them, namely *ERF109* (T14), indicated antiapoptotic effect, where the mean relative cell death value due to OA (20 mM) treatment was significantly the highest in its knockdown VIGS line as compared to the other knockdown lines or controls. The other three genes, namely *ONAC010* (T7), *WRKY53* (T15) and *TFIID5* (T24), indicated apoptotic influence as the mean relative cell death values under treatment were significantly lower in their knocked down VIGS lines than the other knockdown lines or controls. The results of qRT-PCR confirmed the occurrence of knockdown in three out of the four TF genes, namely *ERF109*, *WRKY53* and *TFIID5* (Fig. 2) as the predicated amplicon size of *ONAC010* is short, hence, no specific reverse primer was possibly generated. The results of sqRT-PCR confirmed the co-expression of three PCD-related genes with their respective TFs (Additional file 3: Table S2 & Additional file 8: Figure S5). The PCD-related genes co-expressed with *ERF109*, *WRKY53* and *TFIID5* genes are *Bax Inhibitor 1* (*BI-1*), *apoptosis-inducing factor homolog a-like* (*AIF-a*) and *mildew resistance locus O* (*Mlo*), respectively. sqRT-PCR was also done for the VIGS lines of other TFs and confirmed the occurrence of gene silencing (Additional file 6: Figure S3) albeit the lack of influence during PCD. Silencing was also not tested in VIGS lines of *GTE8* (T2), or *WRKY70* (T16) as no specific reverse primers were possibly generated (Additional file 5: Table S3).

**Table 1:** Description of TF transcripts co-expressed with PCD-related genes knocked down via VIGS and multiple comparisons of the mean relative cell death as responses of tobacco leaf discs following OA (20 mM, pH 7.0) treatment for 24 h as determined by Evans blue staining. Dye released from dead cells was measured at absorbance at 600 nm. Measurements were expressed as relative values with “one” corresponds to the maximum value and others are relative to it. Data are presented as means from two independent experiments with three replicates each. Red boxes indicate lower level of cell death in leaf discs, while blue box indicate higher level of cell death upon knock down of transcript as compared to leaf discs of the wild type non-transformed plant (WT) or those of plants transformed with pRTV2 only

Code	Description	OA treatment	
		0 mM	20 mM
	WT	0.21^C^	0.69^B^
	TRV2 only	0.27^C^	0.63^B^
T1	ethylene-responsive transcription factor 5-like	0.29^C^	0.67^B^
T2	transcription factor gte8-like isoform x2	0.23^C^	0.65^B^
T6	ethylene-responsive transcription factor abr1-like isoform 1	0.24^C^	0.60^B^
T7	nac transcription factor onac010-like	0.27^C^	0.30^C^
T11	light-inducible protein cprf2-like	0.19^C^	0.57^B^
T12	transcription initiation factor tfiid subunit 15b-like isoform x3	0.29^C^	0.59^B^
T13	transcription factor divaricata-like	0.29^C^	0.75^B^
T14	ethylene-responsive transcription factor erf109-like	0.24^C^	1.00^A^
T15	probable wrky transcription factor 53-like	0.17^C^	0.27^C^
T16	probable wrky transcription factor 70-like	0.18^C^	0.65^B^
T17	myb-related protein 305-like	0.32^C^	0.78^B^
T19	ethylene-responsive transcription factor erf109-like	0.30^C^	0.76^B^
T20	nac transcription factor onac010-like	0.27^C^	0.65^B^
T21	ethylene-responsive transcription factor abr1-like isoform 2	0.26^C^	0.79^B^
T23	BED zinc finger	0.22^C^	0.75^B^
T24	transcription initiation factor tfiid subunit 5-like	0.16^C^	0.33^C^
T25	nac transcription factor onac010-like	0.34^C^	0.61^B^
T28	transcription factor spt20 homolog	0.22^C^	0.70^B^
T31	probable wrky transcription factor 23-like	0.23^C^	0.74^B^

Means followed by the same letter are not significantly different by Duncan’s New Multiple Range test (<0.05)

### Performance of Arabidopsis knockout and over-expression lines under salt stress

**Fig. 3:**
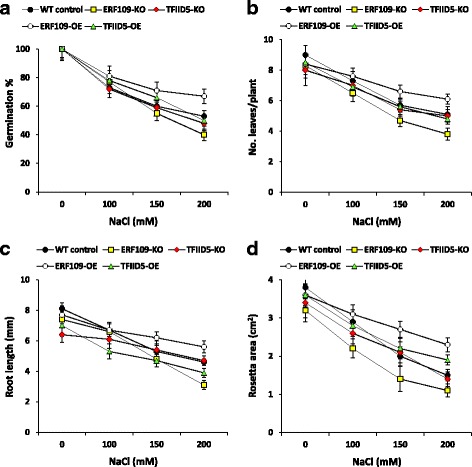
**a** Effect of salt stress on germination percentage (at day 6) of seeds germinated on MS medium supplemented with different concentrations of NaCl (0, 100, 150 or 200 mM) for two selected Arabidopsis T-DNA insertion knockout (KO) mutants and over-expression (OE) lines of genes encoding two TFs, namely ERF109 (atT14) and TFIID5 (atT24), as compared to the WT control (Col). Seeds of the four genotypes were germinated at 0 mM NaCl, left for 2 weeks on MS agar plates, then, transferred to the soil for two more weeks with different salt concentrations. Growth parameters included root length (mm) (**b**), no. leaves per plant (**c**) and rosette area (cm^2^) (**d**). Statistical analysis was performed following the procedure outlined by Gomez and Gomez [28] and multiple comparisons were performed following Duncan’s New Multiple Range test [29]. Standard error bars that do not overlap indicate that the difference between two means is statistically significant

Analogs of two out of the four TF genes (Additional file 9: Table S4), namely *NAC010* and *WRKY53*, have no seeds available at the time of the experiment in Arabidopsis seed stock at the SALK Institute, Genomic Analysis Laboratory (SIGnAL). Genes encoding the other two TFs, e.g., *ERF109*, and *TFIID5*, had seeds available for both T-DNA knockout (KO) and over-expression (OE) lines (Additional file 9: Table S4), hence, used in salt stress experiment after expected expression levels were proven (Additional file 10: Figure S6). Based on PCD assay, the first gene was shown to act as antiapoptotic gene, while the second was shown to act as apoptotic gene. Seeds of the two KO and the two OE lines of *ERF109* and *TFIID5* genes as well as seeds of the WT were left to germinate at different concentrations of NaCl (0, 100, 150 and 200 mM) in order to detect the percentages of germination at day 6 [8]. The results indicated no significant differences among lines up to 150 mM NaCl, while significant differences were scored for germination percentage under 200 mM NaCl (Fig. 3a). The highest significant value was scored for *ERF109-OE* line, while the lowest was scored for *ERF-KO* line. OE and KO of *TFIID5* gene resulted in no significant differences in germination percentages as compared to the WT plant.

In a parallel salt stress experiment, 2-week-old plants of the different genotypes were grown in the soil for two more weeks under different salt treatments. Then, plants were characterized for the mean root length (mm), number of leaves/plant and rosette areas (cm^2^) (Fig. 3b-d). The results for the three parameters indicated the highest significant values for *ERF109-OE* line starting 150 mM NaCl as compared to the WT and the other genotypes, while *ERF109-KO* line showed the lowest. There were no significant differences among *TFIID5-OE*, *TFIID5-KO* and WT plants under different salt concentrations for different characteristics except for rosette area, where *TFIID5-OE* showed significantly higher values as compared to the WT and the two KO mutants.

## Discussion

Ethylene-responsive element-binding factors (ERFs) is a gene family of transcriptional factors that is involved in the adaptation to biotic or abiotic stresses [30–32]. One prior incidence indicated the participation of a member of this family in PCD [19]. The latter incidence is the only support to our data in confirming the role of an ERF gene during PCD. Previous reports indicated that *ERF109* gene (also known as RRTF) responds in Arabidopsis to ethylene (ET) and jasmonic acid (JA) in order to regulate redox homeostasis during both biotic and abiotic stresses [33, 34]. Over-expression of the gene also conferred the enhanced resistance to oxidative stress [35]. Other reports support the finding that *ERF109* gene functions in environmental signal transduction [11]. The gene also regulates lateral root formation in vascular tissues via mediating cross-talking between JA signaling and auxin biosynthesis, where the TF binds to the promoters of *ASA1* and *YUC2* genes, which encode two key enzymes in auxin biosynthetic pathway [36]. Studying *ERF109* KO mutants in Arabidopsis suggested that *ERF109* gene has a positive role in salt-induced lateral root development [36]. We argue that the changes in root architecture might affect ion disequilibrium and Na+/K+ exchange in plants under salt stress, hence, results in the improved tolerance to adverse conditions. The results shown in Additional file 9: Figure S5 also indicates the involvement of *ERF109* gene in the expression of Bax Inhibitor-1 (or *BI-1*) gene. The latter gene inhibits apoptosis induced by Bax and enables the cell to adapt to salt stress by controlling stress-induced reactive oxygen species (ROS) production [8, 37]. Thus, *ERF109* gene might have an additional indirect role in retarding PCD and conferring tolerance against abiotic stresses by regulating the expression of *BI-1* gene.

The results of sqRT-PCR for the KO line of this gene in our study indicated no expression of *ERF109* gene. The gene was reported to have a unique role and share no functions with other ERF family members [36]. The gene was also known to be barely expressed in plant under normal condition in agreement with our results. More recent analysis of this gene in *Aquilaria sinensis* calli indicated that the gene was 4-fold expressed after 24 h of salt stress (150 mM NaCl) as compared to its expression level in the normal condition [38]. However, the gene was not previously studied at earlier time points (2 or 12 h) in order to detect the time where the gene reached the highest level of expression. Up-regulation of the genes encoding TFs likely occurs upstream the genes they regulate, hence, expression of *ERF109* gene likely takes place soon after treatment. This speculation is supported in the present study by RNA-Seq data of tobacco, where the gene was highly expressed after 2 h of OA treatment.

It was previously reported that *ERF109* gene is involved in a number of biological processes [39], of which PCD can now be added. Our results of *ERF109* gene in Arabidopsis under salt stress are in complete harmony with those in tobacco under PCD. Therefore, our hypothesis, that knocking down or knocking out TF that regulate an apoptotic gene(s) might allow the plant to survive longer under adverse conditions, can apply to *ERF109* gene. In other words, we argue that *ERF109* gene has a duel effect under both PCD and salt stress.

## Conclusion

The present work aimed at detecting the role of transcription factors (TFs) concordantly expressed with genes related to programmed cell death (PCD) in conferring salt stress tolerance in plant. RNA-Seq analysis was performed for tobacco (*N. benthamiana*) leaves exposed to oxalic acid (20 mM) to induce PCD. PCD-related genes up-regulated after 2 h of treatment were utilized as landmarks to select TFs with concordant expression. Knockdown mutants via virus induced gene silencing (VIGS) of these TFs were generated in tobacco to detect influence during PCD. Knockout insertion mutants of Arabidopsis as well as over-expression lines of one TF, namely ERF109, analogs to those in tobacco, indicated its involvement in conferring salt stress tolerance in Arabidopsis.

## Additional files

Additional file 1: Table S1. Cluster analysis showing fold change values and description of assembled transcripts of SRA database generated from N. benthamiana leaf discs exposed to oxalic acid (20 mM) for 0, 2, 6, 12 and 24 h. Headings of the selected clusters (1-16) utilized in the study are highlighted with blue color, while headings of the other clusters (A-Z) are highlighted in gray color. (XLSX 286 kb)
Additional file 2: Figure S1. Clusters of co-expressed TFs and PCD-related transcripts of tobacco leaf discs as triggered by OA treatment (20 mM) across time (0, 2, 6, 12 and 24 h). All clusters indicate upregulation after 2 h of treatment. (DOCX 3212 kb)
Additional file 3: Table S2. List of TFs (orange box) co-expressed with PCD-related genes in 10 clusters of gene expression in tobacco. (DOCX 21 kb)
Additional file 4: Figure S2. Fold change values of co-expressed TFs and PCD-related transcripts of tobacco leaf discs as triggered by OA treatment (20 mM) across time (0, 2, 6, 12 and 24 h). All clusters indicate up-regulation after 2 h of treatment. Gene and TF codes refer to those in Additional file 3: Table S2. (DOCX 2987 kb)
Additional file 5: Table S3. Tobacco TFs IDs (T1-T31) and primer names and sequences along with the expected amplicon sizes (bp) to be utilized in constructing pTRV2 vectors (blue boxes) for VIGS, in conducting semi-quantitative RT-PCR (orange boxes) or in both (green boxes). Information for amplifying selected tobacco TFs (T14, T15 and T24) via qRT-PCR (purple boxes) and information for amplifying selected tobacco PCD-related genes (G13, G15 and G18) and Arabidopsis TFs ARF109 (AtT14) and TFIID5 (AtTF24), either knocked out or over-expressed, via sqRT-PCR are shown. Gene codes refer to those indicated in Additional file 3: Table S2. (DOCX 28 kb)
Additional file 6: Figure S3. Semi-quantitative RT-PCR for tobacco VIGS lines of 13 knocked down TFs induced 2 h post oxalic acid treatment (20 mM) as compared to their WT and VIGS line with empty pTRV2 (V2) plants. Amplicon sizes of different genes and primers used are shown in Additional file 5: Table S3. The *Nbactin* gene was used as the house-keeping control. Gene codes refer to those indicated in Additional file 3: Table S2. (DOCX 684 kb)
Additional file 7: Figure S4. Oxalic acid-induced cell death after 24 h as visualized by Evans blue staining (a) and DNA laddering (b) in tobacco WT leaf discs. M = DNA standard (1 kb DNA ladder, Fisher Scientific). (DOCX 5915 kb)
Additional file 8: Figure S5. Semi-quantitative RT-PCR of three PCD-related genes (e.g., G13, G15 and G18) in VIGS lines of the three co-expressed TFs (e.g., T14, T15 and T24, respectively) as compared to those in tobacco wild type (WT) and VIGS line with empty pTRV2 (V2) plants (a). The three PCD-related genes and their co-expressing TFs were induced 2 h after oxalic acid (OA) treatment. Amplicon sizes of different genes and primers used are shown in Additional file 5: Table S3. The *Nbactin* gene was used as the house-keeping control (b). Gene codes refer to those indicated in Additional file 3: Table S2. (DOCX 392 kb)
Additional file 9: Table S4. Tobacco TF IDs in tobacco, their analogues in Arabidopsis and knockout (KO) and over-expression lines along with the links to indicate known function in Arabidopsis database (TAIR, http://www.arabidopsis.org/). (DOCX 17 kb)
Additional file 10: Figure S6. Semi-quantitative RT-PCR for Arabidopsis T-DNA knockout (KO) and over-expression (OE) lines of the two selected TFs, e.g., ERF109, and TFIID5 as compared to their WT plant (Col). Amplicon sizes of different genes and primers used are shown in Additional file 5: Table S3. The *Atactin* gene was used as a house-keeping control. (DOCX 1289 kb)

## Abbreviations

AIF-a: Apoptosis-inducing factor homolog a-like
AP2/ERF: APETALA2/ethylene responsive factor
BI-1: Bax Inhibitor 1
Col: Colombia
CRR: cis-regulatory regions
DREBs: Dehydration-responsive element binding-proteins
ET: Ethylene
JA: Jasmonic acid
KO: Knockout
Mlo: Mildew resistance locus O
NCBI: National Center for Biotechnology Information
OA: Oxalic acid
OE: Over-expression
PCD: Programmed cell death
PDS: Phytoene desaturase
RE: Response elements
SIGnAL: SALK Institute, Genomic Analysis Laboratory
sq RT-PCR: Semi-quantitative RT-PCR
TF: Transcription factors
VIGS: Virus induced gene silencing
