# Supplementary material for: Ethylene responsive transcription factor ERF109 retards PCD and improves salt tolerance in plant
Source: BMC Plant Biol. 2016 Oct 6;16:216. doi: 10.1186/s12870-016-0908-z (PMC5053207; doi:10.1186/s12870-016-0908-z)
Supplement: Additional file 2: Figure S1. — Clusters of co-expressed TFs and PCD-related transcripts of tobacco leaf discs as triggered by OA treatment (20 mM) across time (0, 2, 6, 12 and 24 h). All clusters indicate upregulation after 2 h of treatment. (DOCX 3212 kb) [file 12870_2016_908_MOESM2_ESM.docx]

Figure S1.

Figure S1. Continued
