# Supplementary material for: Ethylene responsive transcription factor ERF109 retards PCD and improves salt tolerance in plant
Source: BMC Plant Biol. 2016 Oct 6;16:216. doi: 10.1186/s12870-016-0908-z (PMC5053207; doi:10.1186/s12870-016-0908-z)
Supplement: Additional file 3: Table S2. — List of TFs (orange box) co-expressed with PCD-related genes in 10 clusters of gene expression in tobacco. (DOCX 21 kb) [file 12870_2016_908_MOESM3_ESM.docx]

Table S2. List of TFs (orange box) co-expressed with PCD-related genes in 10 clusters of gene expression in tobacco.

| PCD-related gene (G)/TF (T) | Transcript ID | Description of gene or TF | Detected domain in deduced aa sequences |
| --- | --- | --- | --- |
| **Cluster 3** | | | |
| G1 | GG79261\|c1_g1_i1 | u3 small nucleolar rna-associated protein 4-like | WD domain, G-beta repeat |
| T1 | GG38260\|c0_g1_i1 | ethylene-responsive transcription factor 5-like | AP2 domain |
| T2 | GG18152\|c5_g2_i1 | transcription factor gte8-like isoform x2 |  |
| G2 | GG63750\|c4_g2_i2 | katanin p80 wd40 repeat-containing subunit b1 homolog (AT5G19620) | WD domain, G-beta repeat |
| **Cluster 4** | | | |
| T3* | GG28630\|c0_g1_i1 | dof zinc finger | Dof domain, zinc finger |
| T4* | GG12229\|c4_g1_i1 | bed zinc family dimerization domain isoform 2 | BED zinc finger |
| T5* | GG82539\|c2_g1_i2 | enhancer of yellow 2 transcription factor homolog isoform 2 |  |
| G3 | GG79836\|c0_g1_i1 | metacaspase-3-like isoform x2 | Caspase domain |
| G4 | GG3719\|c1_g1_i2 | bi1-like partial | Inhibitor of apoptosis-promoting Bax1 |
| G5 | GG59017\|c0_g1_i3 | late blight resistance protein homolog r1a-10-like | NB-ARC domain |
| G6 | GG64878\|c6_g1_i2 | ras-related protein raba2a-like | Miro-like protein |
| G7 | GG5937\|c1_g1_i1 | metacaspase 1 isoform x1 | Caspase domain |
| **Cluster 5** | | | |
| G8 | GG40942\|c0_g1_i3 | probable poly(ADP-ribose) | RCD1-SRO-TAF4 (RST) plant domain |
| T6 | GG33309\|c1_g1_i1 | ethylene-responsive transcription factor abr1-like isoform 1 | AP2 domain |
| T7 | GG52980\|c0_g1_i1 | nac transcription factor onac010-like |  |
| **Cluster 6** | | | |
| G9 | GG59097\|c1_g1_i3 | programmed cell death protein 2-like |  |
| T8** | GG63267\|c5_g1_i2 | PREDICTED: uncharacterized protein LOC102579795 | Zinc finger, C3HC4 type (RING finger |
| T9** | GG43164\|c0_g1_i4 | myb-related protein 306-like | Myb-like DNA-binding domain |
| G10 | GG38146\|c5_g1_i5 | mlo-like protein 11-like (promoting cell death) | Mlo family |
| T10** | GG15719\|c1_g1_i2 | PREDICTED: uncharacterized protein LOC101260987 | Myb-like DNA-binding domain |
| G11 | GG70956\|c4_g1_i3 | sec12-like protein 1-like | WD domain, G-beta repeat |
| G12 | GG28140\|c1_g1_i1 | PREDICTED: uncharacterized protein LOC102589284 | WD domain, G-beta repeat |
| T11 | GG45021\|c4_g1_i4 | light-inducible protein cprf2-like | bZIP transcription factor |
| **Cluster 8** | | | |
| T12 | GG75953\|c2_g1_i2 | transcription initiation factor tfiid subunit 15b-like isoform x3 | Zn-finger in Ran binding protein and |
| T13 | GG69170\|c2_g1_i1 | transcription factor divaricata-like |  |
| T14 | GG20232\|c1_g2_i1 | ethylene-responsive transcription factor erf109-like | AP2 domain |
| G13 | GG3719\|c1_g1_i4 | bax inhibitor-1 family protein | Inhibitor of apoptosis-promoting Bax |
| G14 | GG84247\|c3_g1_i2 | gtp-binding protein yptm2-like | Miro-like protein |
| **Cluster 9** | | | |
| T15 | GG11475\|c3_g1_i3 | probable wrky transcription factor 53-like | WRKY DNA -binding domain |
| T16 | GG68415\|c1_g1_i2 | probable wrky transcription factor 70-like | WRKY DNA -binding domain |
| G15 | GG4666\|c0_g1_i7 | apoptosis-inducing factor homolog a-like |  |
| T17 | GG71144\|c0_g1_i1 | myb-related protein 305-like | Myb-like DNA-binding domain |
| T18** | GG44173\|c1_g1_i1 | probable wrky transcription factor 40-like |  |
| T19 | GG32911\|c0_g1_i1 | ethylene-responsive transcription factor erf109-like | AP2 domain |
| G16 | GG85010\|c2_g1_i1 | wd repeat-containing protein rup2-like | WD domain, G-betabb repeat |
| **Cluster 11** | | | |
| G17 | GG61596\|c2_g1_i1 | late blight resistance protein homolog r1b-16-like | NB-ARC domain |
| T20 | GG52980\|c0_g1_i4 | nac transcription factor onac01-like |  |
| T21 | GG51788\|c1_g3_i3 | ethylene-responsive transcription factor abr1-like isoform 2 |  |
| T22** | GG83566\|c0_g1_i4 | transcription factor bhlh137-like | Helix-loop-helix DNA-binding domain |

| **Cluster 12** | | | |
| --- | --- | --- | --- |
| G18 | GG68778\|c0_g1_i6 | Mlo family | mlo-like protein 3-like |
| T23 | GG28525\|c3_g1_i1 | Os05g0521800 | BED zinc finger |
| T24 | GG8942\|c5_g3_i3 | transcription initiation factor tfiid subunit 5-like | WD40 associated region in TFIID subu |
| **Cluster 15** | | | |
| T25 | GG52980\|c0_g1_i3 | nac transcription factor onac010-like |  |
| G19 | GG19032\|c1_g1_i2 | macpf domain-containing protein nsl1-like | MAC/Perforin domain |
| T26* | GG32960\|c0_g1_i1 | ethylene-responsive transcription factor 4-like | AP2 domain |
| T27* | GG88656\|c0_g1_i1 | ethylene-responsive transcription factor crf4-like |  |
| G20 | GG43397\|c1_g1_i2 | programmed cell death protein 2-like | Programmed cell death protein 2, C- |
| G21 | GG56912\|c0_g2_i3 | plasminogen activator inhibitor 1 rna-binding | Stm1 |
| T28 | GG42426\|c0_g1_i1 | transcription factor spt20 homolog |  |
| G22 | GG6585\|c1_g1_i2 | tmv resistance protein n-like | NB-ARC domain |
| T29** | GG69285\|c0_g1_i1 | probable wrky transcription factor 48-like | WRKY DNA -binding domain |
| **Cluster 16** | | | |
| G23 | GG58302\|c1_g1_i3 | probable beta- -xylosyltransferase irx10l-like | Exostosin family |
| T30** | GG36212\|c0_g1_i1 | transcription factor tga7-like |  |
| T31 | GG87227\|c0_g2_i1 | probable wrky transcription factor 23-like |  |

* Due to technical problems, no primers were synthesized for VIGS or semi-quantitative RT-PCR

**Primers synthesized failed to generate amplicons
