# Supplementary material for: Ethylene responsive transcription factor ERF109 retards PCD and improves salt tolerance in plant
Source: BMC Plant Biol. 2016 Oct 6;16:216. doi: 10.1186/s12870-016-0908-z (PMC5053207; doi:10.1186/s12870-016-0908-z)
Supplement: Additional file 4: Figure S2. — Fold change values of co-expressed TFs and PCD-related transcripts of tobacco leaf discs as triggered by OA treatment (20 mM) across time (0, 2, 6, 12 and 24 h). All clusters indicate up-regulation after 2 h of treatment. Gene and TF codes refer to those in Additional file 3: Table S2. (DOCX 2987 kb) [file 12870_2016_908_MOESM4_ESM.docx]

**Fold change**

**Fold change**

**Fold change**

**Fold change**

**Fold change**

**Fold change**

**Fold change**

**Fold change**

Figure S2.

**Fold change**

**Fold change**

**Fold change**

**Fold change**

**Fold change**

**Fold change**

**Fold change**

**Fold change**

Figure S2. Continued

**Fold change**

Figure S2. Continued
