# Supplementary material for: Ethylene responsive transcription factor ERF109 retards PCD and improves salt tolerance in plant
Source: BMC Plant Biol. 2016 Oct 6;16:216. doi: 10.1186/s12870-016-0908-z (PMC5053207; doi:10.1186/s12870-016-0908-z)
Supplement: Additional file 5: Table S3. — Tobacco TFs IDs (T1-T31) and primer names and sequences along with the expected amplicon sizes (bp) to be utilized in constructing pTRV2 vectors (blue boxes) for VIGS, in conducting semi-quantitative RT-PCR (orange boxes) or in both (green boxes). Information for amplifying selected tobacco TFs (T14, T15 and T24) via qRT-PCR (purple boxes) and information for amplifying selected tobacco PCD-related genes (G13, G15 and G18) and Arabidopsis TFs ARF109 (AtT14) and TFIID5 (AtTF24), either knocked out or over-expressed, via sqRT-PCR are shown. Gene codes refer to those indicated in Additional file 3: Table S2. (DOCX 28 kb) [file 12870_2016_908_MOESM5_ESM.docx]

Table S3. Tobacco TFs IDs (T1-T31) and primer names and sequences along with the expected amplicon sizes (bp) to be utilized in constructing pTRV2 vectors (blue boxes) for VIGS, in conducting semi-quantitative RT-PCR (orange boxes) or in both (green boxes). Information for amplifying selected tobacco TFs (T14, T15 and T24) via qRT-PCR (purple boxes) and information for amplifying selected tobacco PCD-related genes (G13, G15 and G18) and Arabidopsis TFs ARF109 (AtT14) and TFIID5 (AtTF24), either knocked out or over-expressed, via sqRT-PCR are shown. Gene codes refer to those indicated in Table S2.

| Code | Transcript ID in tobacco or locus in Arabidopsis | Primers for VIGS and RT-PCR | Sequence (5`-3`) | Amplicon size of sqRT-PCR or VIGS (bp) |
| --- | --- | --- | --- | --- |
| T1 | GG38260\|c0_g1_i1 | GG38260_c0_g1_i1-RT_F | AAA TGT TCC TAG CCA AAC TCG | 389 |
|  |  | GG38260_c0_g1_i1-VIGS_F | **ACC GAA TTC TCT AGA** CTC TGT AAT GCT TCT TCT CGC* | 300 |
|  |  | GG38260_c0_g1_i1-RT/VIGS_R | **ACC GAG CTC ACG CGT CTC GAG** CAC AAG AGA ATT GCA CTT CAC |  |
| T2** | GG18152\|c5_g2_i1 | GG18152_c5_g2_i1-VIGS-F | **ACC GAA TTC TCT AGA** AAT CAA TTT CAC CTT CCT CAG G | 232 |
|  |  | GG18152_c5_g2_i1-VIGS-R | **ACC GAG CTC ACG CGT CTC GAG** GAG GAT TTG GAG ATG CTG AC |  |
| T3*** | GG28630\|c0_g1_i1 |  | | |
| T4*** | GG12229\|c4_g1_i1 |  | | |
| T5*** | GG82539\|c2_g1_i2 |  | | |
| T6 | GG33309\|c1_g1_i1 | GG33309_c1_g1_i1-RT_F | TGT GCA TGA AAA TCA CCA GG | 358 |
|  |  | GG33309_c1_g1_i1-VIGS_F | **ACC GAA TTC TCT AGA** CCA TCT CTG CAC TCT GAA AAG | 300 |
|  |  | GG33309_c1_g1_i1-RT/VIGS_R | **ACC GAG CTC ACG CGT CTC GAG** CAC ACA AAG CAG CTA GAG TAT G |  |
| T7** | GG52980\|c0_g1_i1 | GG52980_c0_g1_i1-VIGS-F | **ACC GAA TTC TCT AGA** TTT GCC TGC CTT AAT CAT CC | 260 |
|  |  | GG52980_c0_g1_i1-VIGS-R | **ACC GAG CTC ACG CGT CTC GAG** TCA TCC CAA GAA GTA GCC G |  |
| T8**** | GG63267\|c5_g1_i2 | GG63267_c5_g1_i2-RT_F | ACA AGT TCT TTG GAG CAG TAA C | 401 |
|  |  | GG63267_c5_g1_i2-VIGS_F | **ACC GAA TTC TCT AGA** ACC TCT CCC TTG CAA ACA GGA C | 339 |
|  |  | GG63267_c5_g1_i2-RT/VIGS_R | **ACC GAG CTC ACG CGT CTC GAG** TGC AGC TCG AAC TTG GCA AC |  |
| T9**** | GG43164\|c0_g1_i4 | GG43164_c0_g1_i4-RT_F | CGA CAT GGA CTG AGA AAC ATC | 350 |
|  |  | GG43164_c0_g1_i4-VIGS_F | **ACC GAA TTC TCT AGA** GAG TGA GTC TAA TCC CTC TGG | 300 |
|  |  | GG43164_c0_g1_i4-RT/VIGS_R | **ACC GAG CTC ACG CGT CTC GAG** GCT CTT TCT GAT GCT TTA TCC C |  |
| T10**,*** | GG15719\|c1_g1_i2 | GG15719_c1_g1_i2-VIGS-F | **ACC GAA TTC TCT AGA** AGC TTT CAG ATC TCT CCA ATT C | 280 |
|  |  | GG15719_c1_g1_i2-VIGS-R | **ACC GAG CTC ACG CGT CTC GAG** GGA GAA ACA ATG GGT GTG G |  |
| T11 | GG45021\|c4_g1_i4 | GG45021_c4_g1_i4-RT_F | TTT CTG CTT CTC CTT CAG CTT C | 350 |
|  |  | GG45021_c4_g1_i4-VIGS_F | **ACC GAA TTC TCT AGA** CAC TGG TTG TTG ACC TCA C | 300 |
|  |  | GG45021_c4_g1_i4-RT/VIGS_R | **ACC GAG CTC ACG CGT CTC GAG** ATA TTG CCG TTG ACT CTG AAG |  |
| T12 | GG75953\|c2_g1_i2 | GG75953_c2_g1_i2-RT_F | GGG AAC TGT CAT AAC CAC CTG C | 336 |
|  |  | GG75953_c2_g1_i2-VIGS_F | **ACC GAA TTC TCT AGA** GCC ATA TCC CCC ATC ATA TCC | 300 |
|  |  | GG75953_c2_g1_i2-RT/VIGS_R | **ACC GAG CTC ACG CGT CTC GAG** GCT GGA GGT GGT AGA ACA AG |  |
| T13 | GG69170\|c2_g1_i1 | GG69170_c2_g1_i1-RT_F | AAG ATG GAA CAT TGA CTC CAA G | 292 |
|  |  | GG69170_c2_g1_i1-VIGS_F | **ACC GAA TTC TCT AGA** TTG ACT CCA AGT GAC CGA C | 303 |
|  |  | GG69170_c2_g1_i1-RT/VIGS_R | **ACC GAG CTC ACG CGT CTC GAG** TGC TAC GAA TAC GGG AAG G |  |
| T14 | GG20232\|c1_g2_i1 | GG20232_c1_g2_i1-VIGS_F | **ACC GAA TTC TCT AGA** TCG TCA ACC AAT CTT GAA TCT C | 300 |
|  |  | GG20232_c1_g2_i1-VIGS-R | **ACC GAG CTC ACG CGT CTC GAG** AAT GGG CAG CAG AAA TTA GAG |  |
| T15 | GG11475\|c3_g1_i3 | GG11475_c3_g1_i3-VIGS_F | **ACC GAA TTC TCT AGA** TTG TCG TCC AAC AAT TTT GC | 300 |
|  |  | GG11475_c3_g1_i3-VIGS_R | **ACC GAG CTC ACG CGT CTC GAG** CTG TGA CTC CAA CAT GTG G |  |
| T16** | GG68415\|c1_g1_i2 | GG68415_c1_g1_i2-VIGS-F | **ACC GAA TTC TCT AGA** AAC AGT TCC CAC CAT CAA ATC | 214 |
|  |  | GG68415_c1_g1_i2-VIGS-R | **ACC GAG CTC ACG CGT CTC GAG** TCA AGG ATG CGA AGC AAA C |  |
| T17 | GG71144\|c0_g1_i1 | GG71144_c0_g1_i1-RT_F | ATT GAT GGG ATA GCA GAT GAA C | 311 |
|  |  | GG71144_c0_g1_i1-VIGS_F | **ACC GAA TTC TCT AGA** AGC AGA TGA ACC TTG TAT CTT C | 300 |
|  |  | GG71144_c0_g1_i1-RT/VIGS_R | **ACC GAG CTC ACG CGT CTC GAG** GGC AAG AGT TGC AGA TTG AG |  |
| T18**** | GG44173\|c1_g1_i1 | GG44173_c1_g1_i1-RT_R | TTC AGA CCT CTT CGA GTT CC | 345 |
|  |  | GG44173_c1_g1_i1-RT/VIGS_F | **ACC GAA TTC TCT AGA** AGT TGT CTT CAT CAC TTG AGC |  |
|  |  | GG44173_c1_g1_i1-VIGS_R | **ACC GAG CTC ACG CGT CTC GAG** CTT GGG AAA GAT CTC ACG C | 283 |
| T19 | GG32911\|c0_g1_i1 | GG32911_c0_g1_i1-RT_F | CAA AAA TGT CGC AGC CTA AAC | 323 |
|  |  | GG32911_c0_g1_i1-VIGS_F | **ACC GAA TTC TCT AGA** CCT TTA ATT CTG CAA AAG GGA C | 300 |
|  |  | GG32911_c0_g1_i1-RT/VIGS_R | **ACC GAG CTC ACG CGT CTC GAG** TGG AAC GTC GTA TAC AGA CC |  |
| T20 | GG52980\|c0_g1_i4 | GG52980_c0_g1_i4-RT_F | AGA TGC TCC AAT CTC CTA CTT G | 395 |
|  |  | GG52980_c0_g1_i4-VIGS_F | **ACC GAA TTC TCT AGA** GTG ACA AGG GAA TAT TGG TGC | 339 |
|  |  | GG52980_c0_g1_i4-RT/VIGS_R | **ACC GAG CTC ACG CGT CTC GAG** GCC TGC CTT AAT CAT CCC TG |  |
| T21 | GG51788\|c1_g3_i3 | GG51788_c1_g3_i3-RT_F | TTC TCC AAA CCC TCC ATA AAC C | 415 |
|  |  | GG51788_c1_g3_i3-VIGS_F | **ACC GAA TTC TCT AGA** TCT TTT GTC CAG CTC TAG ACC | 338 |
|  |  | GG51788_c1_g3_i3-RT/VIGS_R | **ACC GAG CTC ACG CGT CTC GAG** AAT CAC TAC AAC CAC CGC C |  |
| T22**** | GG83566\|c0_g1_i4 | GG83566_c0_g1_i4-RT_F | CAA GAC TAT GGC TGT CTG TTG | 334 |
|  |  | GG83566_c0_g1_i4-VIGS_F | **ACC GAA TTC TCT AGA** GCT CTA ACA TGA ATG TAG CCT G | 300 |
|  |  | GG83566_c0_g1_i4-RT/VIGS_R | **ACC GAG CTC ACG CGT CTC GAG** GCT TAA TTC TGT GAC CAA CAA C |  |
| T23 | GG28525\|c3_g1_i1 | GG28525_c3_g1_i1-RT_F | AAA CTA CCT GTG CCG CTA C | 326 |
|  |  | GG28525_c3_g1_i1-VIGS_F | **ACC GAA TTC TCT AGA** ACC TAT GCA CAA GTA ATT TCC C | 295 |
|  |  | GG28525_c3_g1_i1-RT/VIGS_R | **ACC GAG CTC ACG CGT CTC GAG** GCA GTT GGT GAT AGA ATA GAC G |  |
| T24 | GG8942\|c5_g3_i3 | GG8942_c5_g3_i3-VIGS_F | **ACC GAA TTC TCT AGA** GAA TAT GAC CTC TAG CGA CAA G | 300 |
|  |  | GG8942_c5_g3_i3-VIGS_R | **ACC GAG CTC ACG CGT CTC GAG** AAA TAG AGA AAG CAG TGG TAG C |  |
| T25 | GG52980\|c0_g1_i3 | GG52980_c0_g1_i3-RT_F | ATG GAG GAG CAG AAG GAG TG | 411 |
|  |  | GG52980_c0_g1_i3-VIGS_F | **ACC GAA TTC TCT AGA** GAA GGA GTG TCC AAA ACC TG | 300 |
|  |  | GG52980_c0_g1_i3-RT/VIGS_R | **ACC GAG CTC ACG CGT CTC GAG** TTA CCC GCT ATG CCA AAA C |  |
| T26*** | GG32960\|c0_g1_i1 |  | | |
| T27*** | GG88656\|c0_g1_i1 |  | | |
| T28 | GG42426\|c0_g1_i1 | GG42426_c0_g1_i1-RT_F | AAT TCT CCA AGA TCT CTC TTG C | 344 |
|  |  | GG42426_c0_g1_i1-VIGS_F | **ACC GAA TTC TCT AGA** ACT TGT TTC TCA AAT GTC GCA G | 300 |
|  |  | GG42426_c0_g1_i1-RT/VIGS_R | **ACC GAG CTC ACG CGT CTC GAG** GCA GCA GCA GCA AAA GAT G |  |
| T29**** | GG69285\|c0_g1_i1 | GG69285_c0_g1_i1-RT_F | TTA CTA CAA GTT CCT CCT CCA C | 321 |
|  |  | GG69285_c0_g1_i1-VIGS_F | **ACC GAA TTC TCT AGA** CAT TAC CAC CAC CAA TGG C | 300 |
|  |  | GG69285_c0_g1_i1-RT/VIGS_R | **ACC GAG CTC ACG CGT CTC GAG** GAT TTG CAT TCA TGA CCA AGA G |  |
| T30**** | GG36212\|c0_g1_i1 | GG36212_c0_g1_i1-RT_F | CTG AAG CTA AGT ATT CCA CAC C | 366 |
|  |  | GG36212_c0_g1_i1-VIGS_F | **ACC GAA TTC TCT AGA** CCT CAA CTC ATC AGC TTC TTC | 300 |
|  |  | GG36212_c0_g1_i1-RT/VIGS_R | **ACC GAG CTC ACG CGT CTC GAG** CGG CTA ATT TAT GCT CTT TGT G |  |
| T31 | GG87227\|c0_g2_i1 | GG87227_c0_g2_i1-RT_F | TGA CGA TGA AGA GAT CGA CAA C | 363 |
|  |  | GG87227_c0_g2_i1-VIGS_F | **ACC GAA TTC TCT AGA** ACA ACG AAT TAG GCG TCG | 297 |
|  |  | GG87227_c0_g2_i1-RT/VIGS_R | **ACC GAG CTC ACG CGT CTC GAG** ACA AGC TAA AAG CTG AGA CTT C |  |
| G13 | GG3719\|c1_g1_i4 | GG3719_c1_g1_i2-RT_F | GCA AGA GAA ACT GTG AAA ACC C | 319 |
|  |  | GG3719_c1_g1_i2-RT_R | AGG CGT ACC GGA AAG ATG AC |  |
| G15 | GG4666\|c0_g1_i7 | GG4666_c0_g1_i7-RT_F | CCT TTG CAT GTT CTT GAG CC | 340 |
|  |  | GG4666_c0_g1_i7-RT_R | GGA GAA AGT GCC AGC AAA AAG |  |
| G18 | GG68778\|c0_g1_i6 | GG68778_c0_g1_i6-RT_F | CCC TGA TAA CGA TAA TCT CCA C | 316 |
|  |  | GG68778_c0_g1_i6-RT_R | GGT TGG AAC GTC TAT CTT TGG |  |
|  | *Nbactin* | NbActin-F | AAG ATA CTC ACA GAA AGA GGC TAC TC | 372 |
|  |  | NbActin-R | GGG AGC TAA TGC AGT AAT TTC CTT |  |
| T14 | GG20232\|c1_g2_i1 | GG20232_c1_g2_i1-RT_F2 | GGC TGC GAC ATT TTT GGA ACC ACG | 190 |
|  |  | GG20232_c1_g2_i1-RT-R2 | TCC TTG GAT CTC TAA TTT CTG CTG CCC |  |
| T15 | GG11475\|c3_g1_i3 | GG11475_c3_g1_i3-RT_F | AGG ACC TTC AAA TCC AGT CTC TGC AC | 196 |
|  |  | GG11475_c3_g1_i3-RT_R | ATC TCC ACA GCC TGT GAC TCC AAC |  |
| T24 | GG8942\|c5_g3_i3 | GG8942_c5_g3_i3-RT_F2 | TGC ACC TTG TTT TCC ACC TTC AAC TG | 199 |
|  |  | GG8942_c5_g3_i3-RT_R2 | TCT CGT TGG AAG TGG TCC TGA TGC |  |
|  | *Nbactin* | NbActin-F2 | TGC CCT CCC ACA TGT CAT TCT TCG | 196 |
|  |  | NbActin-R2 | GTT CCT GCT CAT AGT CTA GGG CTA CG |  |
| AtTF14 (EFR109) | AT4G34410 | AtERF109-RT_F | GGT GCT TTA CAC CAA CAG AGT C | 520 |
|  |  | AtERT109-RT_R | TTT CCC CAA GGT CTT TGC CT |  |
| AtTF24 (TFIID5) | AT5G25150 | AtTFIID5-RT_F | TGA AGG CGT GCT TTC TCC AT | 466 |
|  |  | AtTFIID5-RT_R | TGC ATT ACC CGC TTT GTC CT |  |
|  | *Atactin* (AT3G18780) | AtActin-F | CCA GTG TTG TTG GTA GGC CA | 353 |
|  |  | AtActin-R | GGT TGT ACG ACC ACT AGC GT |  |

* Underlined bold letters include EcoRI/XbaI (**ACC GAA TTC TCT AGA**) and SacI/XhoI (**ACC GAG CTC ACG CGT CTC GAG**) sites for VIGS construction

** Short sequence, thus, no reverse (forward for T18) primers for semi-quantitative RT-PCR were generated

*** Due to technical problems, no primers were synthesized for VIGS or semi-quantitative RT-PCR

**** Primers synthesized failed to regenerate amplicons
