# Supplementary material for: Ethylene responsive transcription factor ERF109 retards PCD and improves salt tolerance in plant
Source: BMC Plant Biol. 2016 Oct 6;16:216. doi: 10.1186/s12870-016-0908-z (PMC5053207; doi:10.1186/s12870-016-0908-z)
Supplement: Additional file 6: Figure S3. — Semi-quantitative RT-PCR for tobacco VIGS lines of 13 knocked down TFs induced 2 h post oxalic acid treatment (20 mM) as compared to their WT and VIGS line with empty pTRV2 (V2) plants. Amplicon sizes of different genes and primers used are shown in Additional file 5: Table S3. The Nbactin gene was used as the house-keeping control. Gene codes refer to those indicated in Additional file 3: Table S2. (DOCX 684 kb) [file 12870_2016_908_MOESM6_ESM.docx]

(a) *Nbactin* gene

WT V2 T1 T6 T11 T12 T13 T17 T19 T20 T21 T23 T25 T28 T31

(b) TFs

WT V2 T1 WT V2 T6 WT V2 T11 WT V2 T12 WT V2 T13

WT V2 T17 WT V2 T19 WT V2 T20 WT V2 T21 WT V2 T23

WT V2 T25 WT V2 T28 WT V2 T31

Figure S3.
