# Supplementary material for: Ethylene responsive transcription factor ERF109 retards PCD and improves salt tolerance in plant
Source: BMC Plant Biol. 2016 Oct 6;16:216. doi: 10.1186/s12870-016-0908-z (PMC5053207; doi:10.1186/s12870-016-0908-z)
Supplement: Additional file 7: Figure S4. — Oxalic acid-induced cell death after 24 h as visualized by Evans blue staining (a) and DNA laddering (b) in tobacco WT leaf discs. M = DNA standard (1 kb DNA ladder, Fisher Scientific). (DOCX 5915 kb) [file 12870_2016_908_MOESM7_ESM.docx]

Figure S4.
