# Supplementary material for: Ethylene responsive transcription factor ERF109 retards PCD and improves salt tolerance in plant
Source: BMC Plant Biol. 2016 Oct 6;16:216. doi: 10.1186/s12870-016-0908-z (PMC5053207; doi:10.1186/s12870-016-0908-z)
Supplement: Additional file 8: Figure S5. — Semi-quantitative RT-PCR of three PCD-related genes (e.g., G13, G15 and G18) in VIGS lines of the three co-expressed TFs (e.g., T14, T15 and T24, respectively) as compared to those in tobacco wild type (WT) and VIGS line with empty pTRV2 (V2) plants (a). The three PCD-related genes and their co-expressing TFs were induced 2 h after oxalic acid (OA) treatment. Amplicon sizes of different genes and primers used are shown in Additional file 5: Table S3. The Nbactin gene was used as the house-keeping control (b). Gene codes refer to those indicated in Additional file 3: Table S2. (DOCX 392 kb) [file 12870_2016_908_MOESM8_ESM.docx]

(a) Co-expressed PCD-related genes

(b) *Nbactin* gene

Figure S5.
