# Supplementary material for: Ethylene responsive transcription factor ERF109 retards PCD and improves salt tolerance in plant
Source: BMC Plant Biol. 2016 Oct 6;16:216. doi: 10.1186/s12870-016-0908-z (PMC5053207; doi:10.1186/s12870-016-0908-z)
Supplement: Additional file 9: Table S4. — Tobacco TF IDs in tobacco, their analogues in Arabidopsis and knockout (KO) and over-expression lines along with the links to indicate known function in Arabidopsis database (TAIR, http://www.arabidopsis.org/). (DOCX 17 kb) [file 12870_2016_908_MOESM9_ESM.docx]

Table S4. Tobacco TF IDs in tobacco, their analogues in *Arabidopsis* and knockout (KO) and over-expression lines along with the links to indicate known function in *Arabidopsis* database (TAIR, http://www.arabidopsis.org/).

| Code | Transcript ID | Description in tobacco | Arabidopsis analog locus | T-DNA insertion and over-expression lines | Website |
| --- | --- | --- | --- | --- | --- |
| T7* | GG52980\|c0_g1_i1 | nac transcription factor onac010-like | AT1G28470 | [SALK_000287](https://www.arabidopsis.org/servlets/TairObject?type=germplasm&id=4510435)  [CS212871](https://www.arabidopsis.org/servlets/TairObject?type=germplasm&id=6530532058) | https://www.arabidopsis.org/servlets/TairObject?id=30291&type=locus |
| T14 | GG20232\|c1_g2_i1 | ethylene-responsive transcription factor erf109-like | AT4G34410 | SALK_150614  CS2102255 | https://www.arabidopsis.org/servlets/TairObject?type=locus&name=At4g34410 |
| T15* | GG11475\|c3_g1_i3 | probable wrky transcription factor 53-like | AT4G23810 | [SALK_034157](https://www.arabidopsis.org/servlets/TairObject?type=germplasm&id=4636745) | https://www.arabidopsis.org/servlets/TairObject?id=128526&type=locus |
| T24 | GG8942\|c5_g3_i3 | transcription initiation factor tfiid subunit 5-like | AT5G25150 | SALK_021380  CS872747 | https://www.arabidopsis.org/servlets/TairObject?name=AT5G25150&type=locus |

* No seeds are available in TAIR for knockout mutant and/or over-expression line to be tested in salt stress experiment
