# Supplementary material for: Ethylene responsive transcription factor ERF109 retards PCD and improves salt tolerance in plant
Source: BMC Plant Biol. 2016 Oct 6;16:216. doi: 10.1186/s12870-016-0908-z (PMC5053207; doi:10.1186/s12870-016-0908-z)
Supplement: Additional file 10: Figure S6. — Semi-quantitative RT-PCR for Arabidopsis T-DNA knockout (KO) and over-expression (OE) lines of the two selected TFs, e.g., ERF109, and TFIID5 as compared to their WT plant (Col). Amplicon sizes of different genes and primers used are shown in Additional file 5: Table S3. The Atactin gene was used as a house-keeping control. (DOCX 1289 kb) [file 12870_2016_908_MOESM10_ESM.docx]

Figure S6.
